# Live-Cell Monitoring of Piecemeal Chloroplast Autophagy

**DOI:** 10.21769/BioProtoc.5482

**Published:** 2025-11-05

**Authors:** Masanori Izumi, Sakuya Nakamura, Shinya Hagihara

**Affiliations:** RIKEN Center for Sustainable Resource Science (CSRS), Wako, Japan

**Keywords:** *Arabidopsis*, Autophagy, Chloroplast, Confocal microscope, Fluorescent proteins, Leaf, Live-cell imaging

## Abstract

When plants undergo senescence or experience carbon starvation, leaf cells degrade proteins in the chloroplasts on a massive scale via autophagy, an evolutionarily conserved process in which intracellular components are transported to the vacuole for degradation to facilitate nutrient recycling. Nonetheless, how portions of chloroplasts are released from the main chloroplast body and mobilized to the vacuole remains unclear. Here, we developed a method to observe the autophagic transport of chloroplast proteins in real time using confocal laser-scanning microscopy on transgenic plants expressing fluorescently labeled chloroplast components and autophagy-associated membranes. This protocol enabled us to track changes in chloroplast morphology during chloroplast-targeted autophagy on a timescale of seconds, and it could be adapted to monitor the dynamics of other intracellular processes in plant leaves.

Key features

• This protocol enables real-time monitoring of chloroplast morphology in living *Arabidopsis* leaves.

• The method is based on confocal microscopy of transgenic plants that express fluorescent protein markers for specific organelles or suborganellar compartments.

• We used this protocol to monitor the piecemeal autophagic degradation of chloroplasts, but it could also be extended to other intracellular phenomena.

## Graphical overview


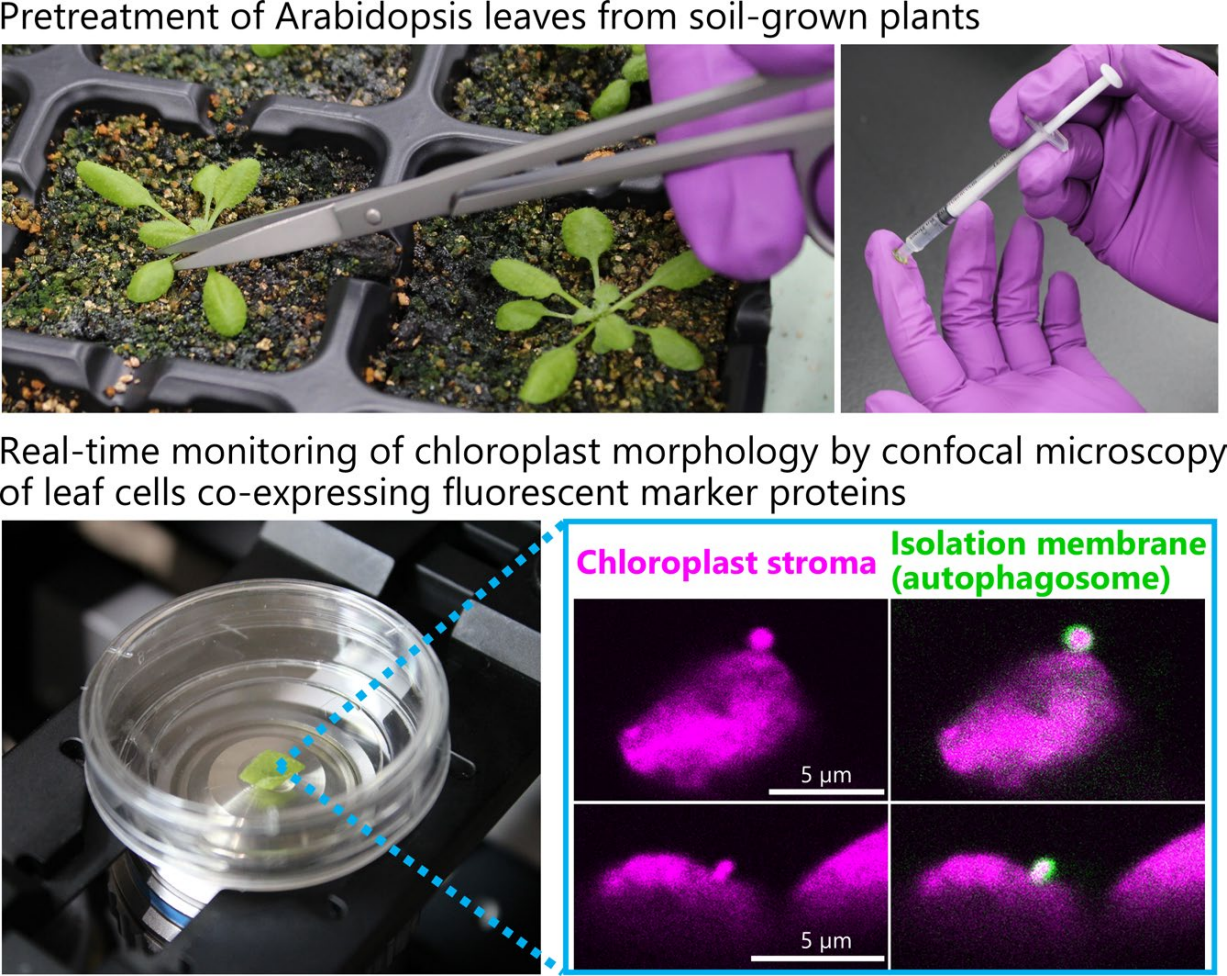


## Background

Many of the nutrients in leaves are found in chloroplasts, where they are incorporated into the protein complexes involved in photosynthesis [1]. During senescence or starvation, chloroplast proteins are degraded on a massive scale, releasing amino acids for reuse in developing organs or for acclimation to starvation conditions. Our previous studies revealed that autophagy is one route for the degradation of chloroplast components [2,3].

Autophagy is a ubiquitous process in eukaryotic cells by which proteins and organelles are transported into the vacuole or lysosomes for degradation. The most extensively characterized form of autophagy is macroautophagy, during which nascent closed-membrane structures termed isolation membranes (or phagophores) elongate to form double membrane–bound autophagosomes that sequester cytoplasmic components [4]. The outer membrane of the autophagosome fuses with the vacuolar/lysosomal membrane, leading to the digestion of the contents of these membrane-bound structures (termed autophagic bodies) by vacuolar/lysosomal lytic activities [4]. When leaves of *Arabidopsis (Arabidopsis thaliana*) or rice (*Oryza sativa*) expressing chloroplast stroma-targeted fluorescent proteins were incubated in the dark with concanamycin A, which suppresses vacuolar lytic activity, small bodies (~1 μm in diameter) containing chloroplast stromal components accumulated in the vacuolar lumen [2,3,5]. Such structures were also detected by immunoelectron microscopy in the leaves of wheat (*Triticum aestivum*) and *Arabidopsis* [2,6]. This vacuolar accumulation of chloroplast-derived bodies did not occur in mutants of macroautophagy-related genes [2,3]. These previous studies suggest that chloroplast proteins are transported into the vacuole as autophagosomal cargo for degradation. However, they describe only the vacuolar accumulation of potentially autophagosomal cargo, which is the final step in macroautophagy. How portions of the chloroplast are released from the main chloroplast as spherical bodies and then mobilized into the vacuole remains to be clarified.

To examine this process, we prepared transgenic *Arabidopsis* plants that stably co-accumulate two different fluorescent proteins visualizing chloroplast stroma and specific chloroplast membranes or autophagic membranes, and established preparation steps of *Arabidopsis* leaves for live-cell monitoring of chloroplast morphology via confocal microscopy. The developed method allowed us to find that chloroplast fragmentation occurs in synchrony with the maturation of the chloroplast-associated isolation membrane [7]. The method could be modified to monitor various intracellular processes associated with chloroplasts in leaf mesophyll cells. Here, we describe the protocol for observing and tracking chloroplast morphology in mature *Arabidopsis* leaves using fluorescent protein markers and confocal laser-scanning microscopy.

## Materials and reagents

**Biological materials**

1. *Arabidopsis thaliana* (L.) Heynh., accession Columbia-0 (Col-0)

2. *Arabidopsis* plants harboring *ProRBCS2B:RBCS2B-mRFP* [7]; *RBCS2B* (AT5G38420) encodes Rubisco small chain 2B, a stromal marker

3. *Arabidopsis* plants harboring *ProRBCS2B:RBCS2B-sGFP (S65T)* [2]

4. *Arabidopsis* plants harboring *ProKEA1:KEA1-mRFP* [7] and *ProRBCS2B:RBCS2B-sGFP; KEA1* (AT1G01790) encodes K^+^ EFFLUX ANTIPORTER 1, a marker of the chloroplast inner envelope

5. *Arabidopsis* plants harboring *ProTOC64:TOC64-mRFP* [7] and *ProRBCS2B:RBCS2B-sGFP; TOC64* (AT3G17970) encodes TRANSLOCON AT THE OUTER MEMBRANE OF CHLOROPLASTS 64-III, a chloroplast outer-envelope marker

6. *Arabidopsis* plants harboring *ProATPC1:ATPC1-tagRFP* [7] and *ProRBCS2B:RBCS2B-sGFP; ATPC1* (AT4G04640) encodes the ATP synthase gamma subunit, a thylakoid membrane marker

7. *Arabidopsis* plants harboring *ProUBQ10:EGFP-ATG8a* [8] and *ProRBCS2B:RBCS2B-mRFP; ATG8a* (AT4G21980) encodes AUTOPHAGY8a, which is an isolation membrane marker

**Reagents**

1. 2-(*N*-morpholino)ethanesulfonic acid (MES) monohydrate (Nacalai, catalog number: 21623-26)

2. NaOH (Nacalai, catalog number: 31511-05; product format: 1 M in ultrapure water)

**Solutions**

1. 0.5 M MES-NaOH stock solution, pH 5.5 (see Recipes)

2. 10 mM MES-NaOH, pH 5.5 (MES buffer) (see Recipes)

**Recipes**

**1. 0.5 M MES-NaOH stock solution, pH 5.5**

**Table:** 

Reagent	Final concentration	Quantity or volume
MES monohydrate	0.5 M	21.33 g
1 M NaOH		As required to adjust the pH to 5.5 (approximately 13 mL)
Ultrapure water		Make up to 200 mL

Store in a refrigerator.

**2. 10 mM MES-NaOH, pH 5.5 (MES buffer)**

The 0.5 M MES-NaOH (pH 5.5) stock solution should be diluted 50-fold with ultrapure water immediately before use.

**Laboratory supplies**

1. Potting soil (Sakata Super Mix A, Sakata Seed Corporation)

2. Fine-grain vermiculite (Nittai, catalog number: G20-60L)

3. Liquid fertilizer (Hyponex undiluted solution, Hyponex Japan)

4. Plastic tray (e.g., AS ONE Corporation, catalog number: 1-4617-02)

5. Plastic cell tray (25 cells, each 49 mm × 49 mm × 56.5 mm) (Meiwa, catalog number: 4402)

6. Stainless steel scissors

7. Stainless steel tweezers

8. Glass-bottom culture dish (Matsunami, catalog number: D11140H)

9. Round cover glass (Fisher Scientific, catalog number: 12-545-102)

10. 1 mL syringe (Terumo, catalog number: SS-01T)

11. 1.5 mL microcentrifuge tube

12. 200 μL micropipette

13. 200 μL wide-bore pipette tips (BMBio ECO, catalog number: BMT-200W)

14. 12-well cell culture plates (Violamo, catalog number: VTC-P12)

15. Plastic wrap

16. Aluminum foil

## Equipment

1. Plant growth chamber equipped with LED lamps (Nippon Medical & Chemical Instruments, catalog number: LPH-411PFDT-SPC)

2. Confocal laser-scanning microscope (Carl Zeiss, model: LSM 900)

3. Water-immersion objective lens (Carl Zeiss, model: C-Apochromat 63×/1.20 W Korr)

4. Water-immersion objective lens (Carl Zeiss, model: C-Apochromat 40×/1.20 W Korr)

5. Refrigerator (Nihon Freezer, model: UKS-3610DHC)

6. Temperature-controlled incubator (e.g., AS ONE Corporation, model: FCI-280HG)

## Software and datasets

1. ZEN software (Carl Zeiss, version 3.3)

2. ZEN lite software (Carl Zeiss, version 3.8)

3. Imaris software (Oxford Instruments, version 9.1)

## Procedure

**A. Growth of *Arabidopsis* plants**

**A1. Cold stratification treatment**

1. Place *Arabidopsis* seeds into a 1.5 mL microcentrifuge tube. The number of seeds is approximately four times the number of plants required for each analysis.

2. Add 500–1,000 μL of ultrapure water to the tube.

3. Cover the tube with aluminum foil to exclude light.

4. Place the tube in a refrigerator at 4 °C for 2–4 days.

**A2. Seed sowing and plant cultivation**

**Figure 1.**
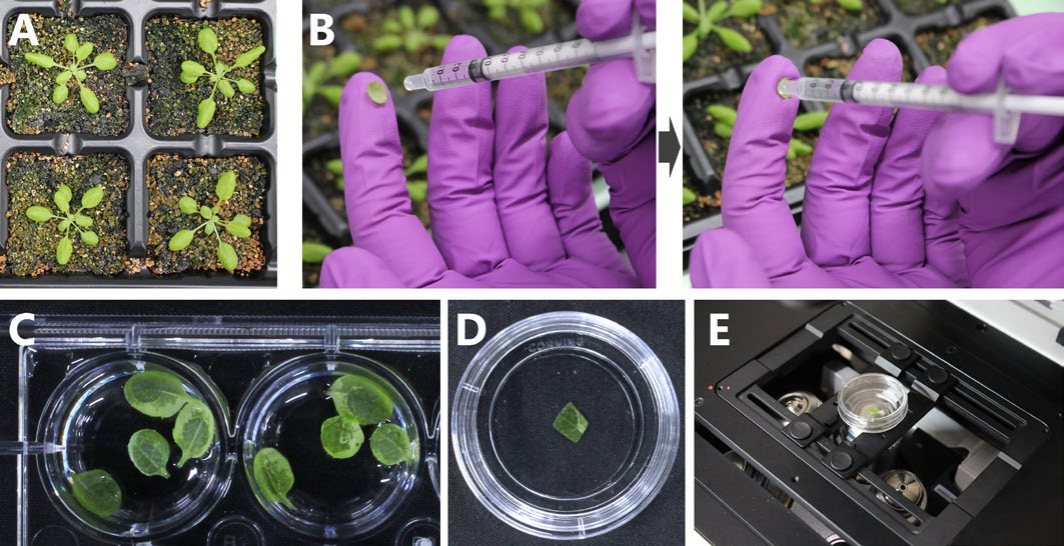
Sugar starvation treatment and confocal microscopy observation of living *Arabidopsis* leaves. (A) *ProRBCS2B:RBCS2B-mRFP* plants expressing a fluorescently tagged chloroplast stromal marker protein were grown for 21 days in soil. (B) Infiltration of MES buffer into the second rosette leaf of a 21-day-old plant. The excised leaf is placed on a fingertip with its abaxial surface facing upward (left). The tip of a 1-mL syringe is used to infiltrate the MES buffer into the leaf with moderate pressure (right). (C) Infiltrated leaves are incubated in a 12-well cell culture plate in the dark. (D) After incubation, the edge of a leaf is removed, placed in a glass-bottom dish, and covered with a round cover glass. (E) The dish is placed on an inverted confocal microscopy system (Zeiss LSM 900) for observation.

1. Mix the potting soil and vermiculite in a 1:1 ratio.

2. Fill individual cells of a plastic cell tray with the prepared soil.

3. Place the plastic cell tray into the plastic tray.

4. Shower tap water onto the soil surface without allowing it to overflow. Repeat the watering process until the soil is completely moist; 10 watering passes are typically sufficient.

5. Discard the outflow water from the bottom of the cell tray.

6. Transfer the stratified seeds from the tube to the soil surface using a 200 μL micropipette fitted with a 200 μL wide-bore pipette tip. We normally sow approximately four seeds in each individual cell.

7. Cover the cell tray with plastic wrap.

8. Place the plastic tray containing the wrapped cell tray into a growth chamber (23 °C, 12/12 h light/dark photoperiod, 90–130 μmol·m^-2^·s^-1^ photon flux density) for germination.

9. Remove the plastic wrap when most of the seeds have germinated, typically 2–4 days after sowing.

10. Remove the smaller plants and equalize the number of plants (to one or two plants) in individual cells approximately 14 days after sowing.

11. Grow plants for 20–24 days after sowing (Figure 1A) and use their second rosette leaves for the following steps. Water with tap water twice a week. Add Hyponex solution (1:2,000 dilution) to the tap water once a week to prevent mineral nutrient deficiencies.

**B. Pretreatment of leaves**

*Note: Here, we describe the pretreatment used to observe morphological changes in chloroplasts in response to sugar starvation. This treatment could be replaced by other treatments, depending on the purpose of the experiments.*

1. Add 1 mL of MES buffer to each well of a 12-well cell culture plate.

2. Using stainless steel scissors, excise the second rosette leaf from each soil-grown plant at the end of a 12-h dark period.

**Critical:** Timing the initiation of dark incubation is critical for the induction of sugar starvation in *Arabidopsis* leaves. Because they accumulate starch during the daytime, it is best to collect leaves for incubation at the end of the dark period when most of this starch has been consumed. All subsequent steps in the protocol should be performed under dim light.

3. Place the leaf on a fingertip using stainless steel tweezers (Figure 1B).

4. Flip the leaf to expose its abaxial surface (Figure 1B, left panel).

5. Remove the air from a 1-mL plastic syringe by repeatedly drawing and ejecting MES buffer. Three to four repetitions are typically sufficient. Then, draw approximately 300 μL of MES buffer into the syringe.

6. Gently press the tip of the syringe onto the abaxial leaf surface and infiltrate the MES buffer into the leaf with moderate pressure by pushing the plunger (Figure 1B, right panel). Less than 10 μL is sufficient for a second rosette leaf.

**Caution:** Intense pressure caused by rapidly pushing the plunger may cause the infiltration solution to scatter.

7. Place the infiltrated leaves into a well of the cell culture plate (Figure 1C).

8. Place the plate in a temperature-controlled incubator at 23 °C. Leave the lid of the plate slightly open for ventilation.

9. Incubate the plate in darkness for 5–8 h. This treatment induces sugar starvation and activates the autophagic degradation of chloroplasts.

*Note: This infiltration treatment also deaerates the leaves, improving the resolution of fluorescent signals during confocal microscopy. When obtaining a snapshot of fluorescent signals in leaf cells, such as the localization of a fluorescent protein or the number of fluorescent protein–labeled organelles, ultrapure water can be used for deaeration, followed by immediate observation with confocal microscopy. For time-lapse monitoring of living leaf cells, the use of a mildly acidic buffer is likely suitable. Although we do not examine how the difference in extracellular pH affects intracellular phenomena, we once observed the inhibition of chloroplast-targeting autophagy when the leaves were accidentally incubated in a pH-unadjusted solution. The infiltration treatment also helps to provide a uniform supply of chemicals to the leaves. For example, we used this infiltration treatment to supply concanamycin A to Arabidopsis leaf mesophyll cells, which stabilized the chloroplast-derived bodies transported by autophagy in the vacuolar lumen [2,5]. Therefore, in this protocol, we infiltrated the MES buffer that does not contain concanamycin A to monitor how portions of chloroplasts are released as small bodies and then transported into the vacuole by autophagy.*

**C. Confocal microscopy observation of chloroplast morphology**

**Figure 2.**
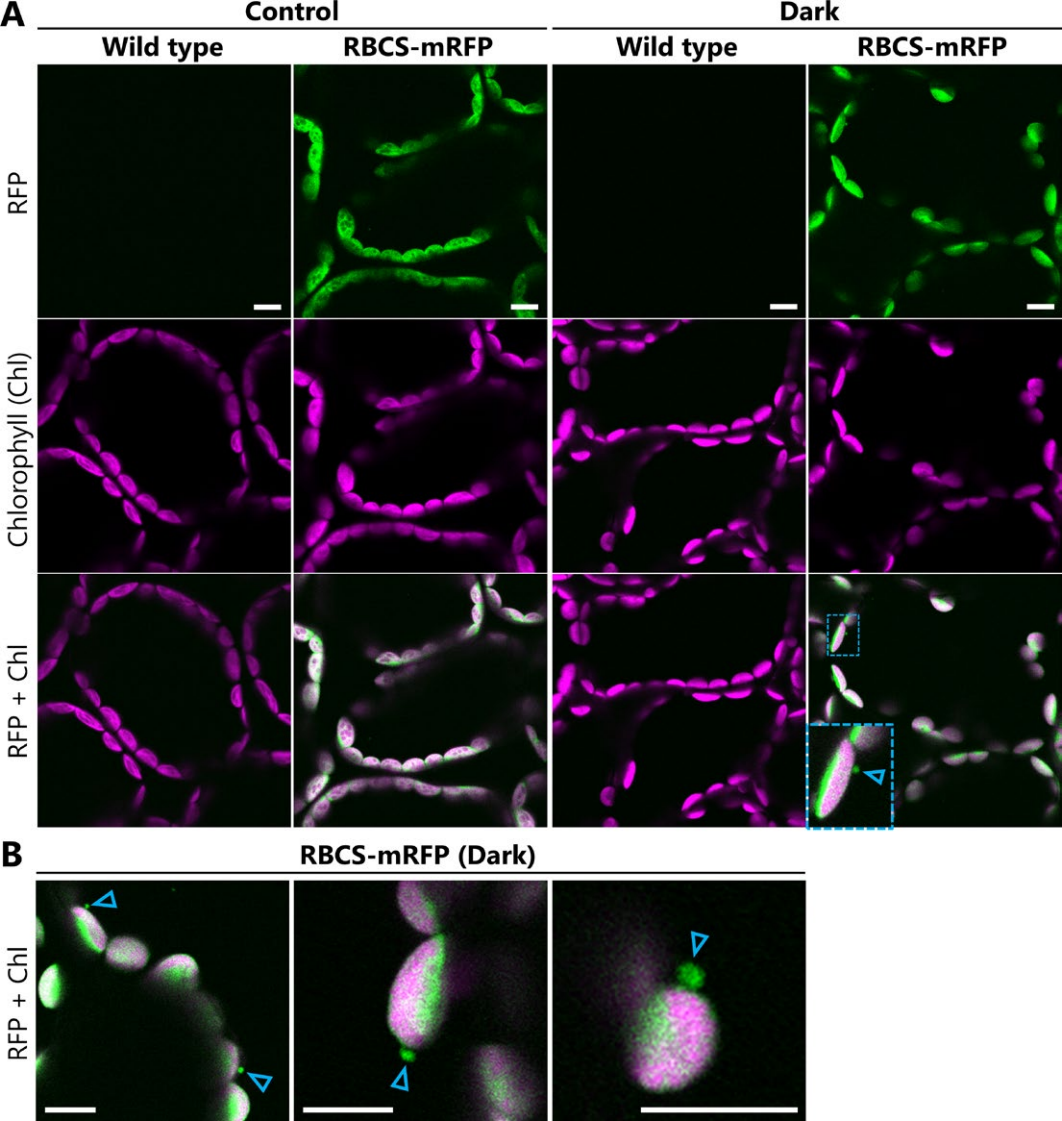
Observations of chloroplast budding structures in *Arabidopsis* leaves exposed to sugar starvation treatment. (A) Confocal microscopy images of mesophyll cells from leaves incubated in the dark for 5–8 h from dawn (Dark) or from leaves excised immediately before observations from plants grown in the light (Control). Second rosette leaves were harvested from soil-grown, 21-day-old wild-type or *ProRBCS2B:RBCS2B-mRFP* (RBCS-mRFP) plants. The area surrounded by blue dashed lines is magnified in the bottom left corner of the image. (B) Additional images of chloroplast budding structures visualized using RBCS-mRFP in leaves exposed to the dark treatment described in (A). Arrowheads indicate the chloroplast budding structures. Green, RFP; magenta, chlorophyll (Chl) fluorescence. Laser power settings: 1.0% for 561 nm; 0.5% for 640 nm. Scale bars, 10 μm.

1. Excise the outer edge of the pretreated leaf with stainless steel scissors immediately before observation.

2. Place the resulting leaf fragment into the glass-bottom dish so that its adaxial surface is in contact with the glass bottom.

3. Place approximately 200 μL of MES buffer around the leaf fragment in the dish.

4. Gently place a round cover glass onto the leaf fragment. The MES buffer should pull the cover glass down onto the leaf fragment to form a seal (Figure 1D). If the leaf fragment is not surrounded by MES buffer or large bubbles are present, the adaxial surface of the leaf fragment does not closely adhere to the glass bottom.

5. Place the glass-bottom dish onto the stage of the confocal laser-scanning microscope (Figure 1E). Use an inverted confocal system for this protocol.

6. Capture the fluorescence signals of each fluorescent marker protein. An example of the microscope settings used to detect GFP, mRFP, and chlorophyll fluorescence is shown in Table 1. Figure 2A shows the fluorescence signals of RBCS-mRFP and chlorophyll in leaves from wild-type and *ProRBCS2B:RBCS2B-mRFP* plants exposed to sugar starvation treatment (dark) or left untreated (control). Untreated leaves were excised from 21-day-old plants grown in the growth chamber immediately before observation and treated as described in steps B3–B6 and C1–C5. The magnified image in Figure 2A and the images in Figure 2B show examples of chloroplast budding structures visualized using RBCS-mRFP in leaves from the dark treatment.

*Note: The observation settings can be modified for different equipment and sample types. We recommend observing wild-type* Arabidopsis *plants that do not express fluorescent proteins to distinguish the fluorescent protein signals from the false signals derived from cellular structures (Figure 1A). Likewise, when leaves are exposed to treatments such as the sugar starvation treatment described here, such false signals should be ruled out by observing wild-type plants exposed to the same treatments, as stresses and related cellular damage can enhance the autofluorescence of cellular structures. For instance, photodamaged chloroplasts in the cytoplasm and chloroplasts undergoing digestion in the vacuole emit faint, broad signals in addition to the chlorophyll-specific signal [9]. When observing plants that express two different fluorescent proteins, also check the plants that express each protein individually to confirm that fluorescence crosstalk does not occur (Figure 2A).*

**Table 1.** Confocal microscope (LSM 900) settings used to detect fluorescent protein and chlorophyll signals in this study

Type of fluorescent signal	Excitation wavelength	Detection wavelength
Green fluorescent protein (GFP)	488 nm	410–546 (or 550) nm
Red fluorescent protein (RFP)	561 nm	550–640 nm
Chlorophyll	488 or 640 nm	656–700 nm

7. Observe *Arabidopsis* leaves expressing the fluorescently labeled stromal marker protein together with markers of other cellular components to determine whether the chloroplast budding structures contain or interact with other compartments.

**Figure 3.**
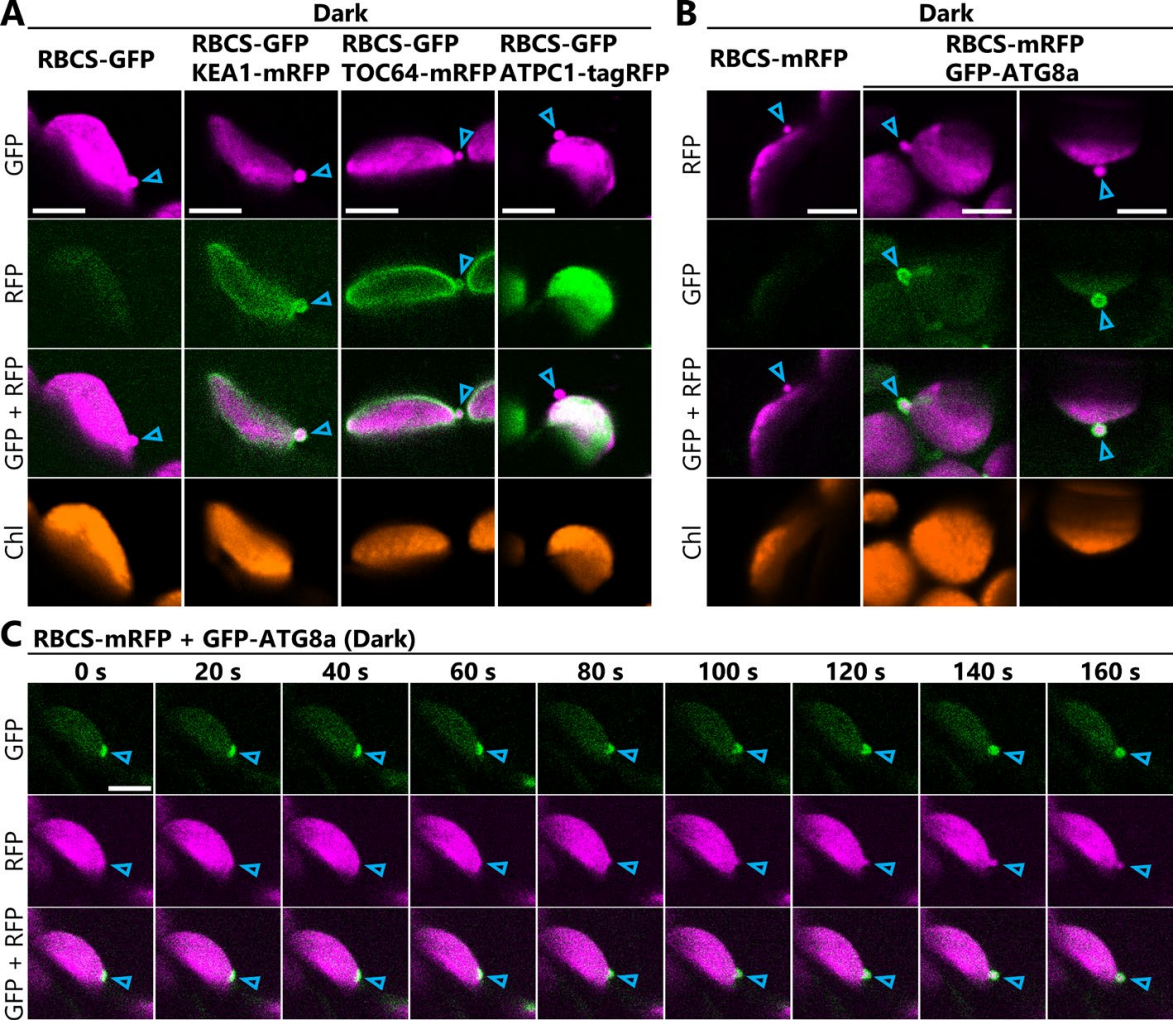
Monitoring chloroplast budding structures in living *Arabidopsis* leaves co-expressing two different fluorescent marker proteins. (A) Confocal microscopy images of mesophyll chloroplasts from second rosette leaves incubated in the dark for 5–8 h from dawn (Dark) at 23 days after sowing. *Arabidopsis* plants harbored the chloroplast stromal marker *ProRBCS2B:RBCS2B-sGFP* (RBCS-GFP) either alone or together with the chloroplast inner-envelope marker *ProKEA1:KEA1-mRFP* (KEA1-mRFP), the chloroplast outer-envelope marker *ProTOC64:TOC64-mRFP* (TOC64-mRFP), or the thylakoid membrane marker *ProATPC1:ATPC1-tagRFP* (ATPC1-tagRFP). Among the three RFP proteins, KEA1-mRFP exhibited the weakest signal, and the most sensitive microscope settings were required to detect it clearly. Leaves from RBCS-GFP plants and plants with both RBCS-GFP and KEA1-mRFP were observed using the same settings to ensure that there was no RBCS-GFP signal crosstalk in the channel for RFP. Laser power settings: 0.8% for 488 nm; 7.0% (ATPC1-tagRFP) or 9.0% (TOC64-mRFP and KEA1-mRFP) for 561 nm. Arrowheads indicate chloroplast budding structures. Green, RFP; magenta, GFP; orange, chlorophyll (Chl) fluorescence. (B) Confocal microscopy images of mesophyll chloroplasts from leaves harboring *ProRBCS2B:RBCS2B-mRFP* (RBCS-mRFP) alone or together with the isolation membrane marker *ProUBQ10:EGFP-ATG8a* (GFP-ATG8a) after the sugar starvation treatment described in (A). Arrowheads indicate chloroplast budding structures. Green, GFP; magenta, RFP; orange, Chl fluorescence. Laser power settings: 1.0% for 488 nm; 1.8% for 561 nm. (C) Still frames from a time-lapse video (Video 1) monitoring the formation of a chloroplast budding structure concurrently with the maturation of an autophagosome. The times displayed above the images indicate the elapsed time from the beginning of the video. Green, GFP; magenta, RFP. Laser power settings: 1.2% for 488 nm; 1.0% for 561 nm. Arrowheads indicate the site at which the isolation membrane contacts the chloroplast. Scale bars, 5 μm.

Figure 3A shows images obtained from leaves expressing the chloroplast stromal marker RBCS-GFP together with the chloroplast inner-envelope marker KEA1-mRFP, the chloroplast outer-envelope marker TOC64-mRFP, or the thylakoid membrane marker ATPC1-tagRFP. The images reveal that the chloroplast budding structures visualized with RBCS-GFP contain chloroplast envelope proteins but not thylakoid membrane proteins. Figure 3B shows images obtained from leaves expressing the chloroplast stromal marker RBCS-mRFP together with the isolation membrane marker GFP-ATG8a. These images reveal that the chloroplast budding structures are surrounded by isolation membranes. These results are consistent with the findings of our previous study, which also used this protocol [7].

8. Use the *Time Series* function in the *Acquisition* tab of ZEN software for time-lapse monitoring. To monitor the formation of chloroplast budding structures, locate the chloroplast-associated isolation membrane labeled by GFP-ATG8a (Figure 3C, left panel) through live scans of the wide field without cropping the scan area. Then, narrow the scan area until the isolation membrane is magnified and begin the time-lapse imaging. Figure 3C and Video 1 show an example of a chloroplast budding structure forming concurrently with the development of the isolation membrane.

**Critical:** We sequentially detected the GFP, RFP, and chlorophyll signals using two different tracks to minimize crosstalk among the different fluorescence signals. The first track detects the GFP and chlorophyll signals simultaneously, while the other detects the RFP signal. Select *Switch track every line* in the *Imaging Setup* box of ZEN software to minimize the time lag between the tracks.

**Video 1.**
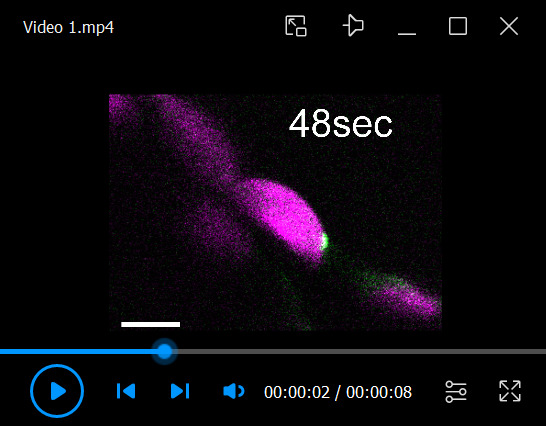
Time-lapse video showing the formation of a chloroplast budding structure concurrently with the development of an isolation membrane. The second rosette leaf from a 21-day-old plant harboring the chloroplast stromal marker *ProRBCS2B-RBCS2B-mRFP* and the isolation membrane marker *ProUBQ10:EGFP-ATG8a* was incubated in the dark for 7 h from dawn and then observed with a Zeiss LSM 900 confocal microscope. Images acquired every 2 s are displayed at 10 frames/s. This video was used to generate the images in Figure 3C. Green, GFP; magenta, RFP. The video only shows the merged channels. Scale bar, 5 μm.

## Data analysis

**A. Measurement of fluorescence intensity in chloroplasts and chloroplast budding structures**

1. Open the image file in ZEN lite software.

2. Select the *Graphics* tab located below the image.

3. Select the *Draw Spline Contour* button.

4. Draw a contour surrounding the region of interest (main chloroplast body, the periphery of a chloroplast, and/or a chloroplast budding structure).

5. Select the *Measure* tab on the left side of the image.

6. Copy the mean fluorescent intensities from the regions of interest.

*Note: This analysis was applied to 30–33 chloroplast budding structures and their associated chloroplasts or chloroplast envelopes from the eight individual plants shown in Figure 2, figure supplement 1 in [7].*

**B. Measurement of chloroplast area and chloroplast budding structures**

1. Open the image file in Imaris software.

2. Select the *Add new Surface* function.

3. Follow the surface creation steps in the *Create* tab. The *Threshold (Absolute intensity)* needs to be optimized as the visible fluorescent signals of the chloroplast stroma–targeted fluorescent protein are recognized as surfaces.

4. The main chloroplast body and the associated budding structure are typically detected as a single surface structure. The area of the budding structure needs to be separated manually. Select the *Surface* button under the *Settings* tab, and then select the *Edit* tab.

5. Select the surface of a chloroplast that is forming a budding structure in the image.

5. Shift-click the point to be separated; the separating line will appear.

6. Select the *Cut Surface* button to divide a surface along the line.

7. Select *Specific Values* and *Area* in the *Detailed* tab under the *Statistics* tab to copy or export the area of the selected surface.

*Note: This analysis was used to track the areas of chloroplast budding structures in Figure 6 and Figure 6, figure supplement 1 in [7]. Imaris is optimized for the analysis of three- or four-dimensional images. This analysis can be used to measure the volumes of structures of interest. Volume values can be selected in the* Detailed *tab. We measured the volume of chloroplasts visualized with RBCS-mRFP in Figure 1, figure supplement 2 in [7]. Tutorials for Imaris software can be found on the provider’s website.*

## Validation of protocol

Structures similar to those shown in Figures 2 and 3, as well as Video 1, were observed in leaves from 2–3 independent plants. This protocol has been used and validated with multiple independent replicates in the following research article:

• Izumi et al. [7] Autophagosome development and chloroplast segmentation occur synchronously for piecemeal degradation of chloroplasts. *eLife* (Figures 2, 6, and 9 and accompanying supplemental figures).

## General notes and troubleshooting

1. This protocol can be modified to monitor other intracellular phenomena in plant leaves. However, transgenic plants harboring fluorescent protein markers for the intracellular structures of interest are required.

2. Please note that the dense accumulation of fluorescent proteins and their aggregates may create artificial structures.

3. Continued observation of an individual leaf sample over time may eventually inhibit biological phenomena, including the formation of chloroplast budding structures. The leaf fragment in the glass-bottom dish may experience stress, including pressure, because it is sandwiched between the bottom of the dish and the round cover glass. Repeated exposure to the excitation lasers can also cause phototoxicity. To minimize the effects of such unavoidable stresses, prepare multiple leaf samples and periodically replace the sample being observed. We do not typically observe an individual leaf sample for longer than 15 min.
